# Role of 0.5 M mannitol as an adjuvant with lidocaine with or without epinephrine for inferior alveolar nerve block: A randomized control trial

**DOI:** 10.4317/jced.55583

**Published:** 2019-06-01

**Authors:** Pranshu-Kumar Pathak, Awadhesh-Kumar Singh, Sudhanshu Agrawal, Dipti Singh, Dhananjay-Kumar Mali, Uday Kumar

**Affiliations:** 1 MDS, Sr. Lecturer, Dept. of Oral Surgery, Purvanchal Institute of Dental Sciences, Gorakhpur, U.P., India; 2 MDS, Professor, Dept. of Periodontology, Chandra Dental College & Hospital, Barabanki, U.P., India; 3 MDS, Asso. Professor, Dept. of Periodontology, Chandra Dental College & Hospital, Barabanki, U.P., India; 4 MDS, Asso. Professor, Dept. of Oral Medicine, Chandra Dental College & Hospital, Barabanki, U.P., India; 5 BDS, Jr. Resident, Dept. of Periodontology, Chandra Dental College & Hospital, Barabanki, U.P., India

## Abstract

**Background:**

The most commonly used local anesthetic in dentistry is lidocaine. For decades, mannitol is the most widely used agent in the management of raised intracranial pressure and as prophylaxis against acute renal failure surgeries.

**Material and Methods:**

120 patients were randomly divided into four groups, 30 patients in each group. Group A was administered 2% lidocaine with 1:80000 epinephrine; group B, 2% lidocaine with 1:80000 epinephrine and 0.5 M mannitol; group C, 2% lidocaine and 0.5 M mannitol; and group D (control group), 2% lidocaine for achieving local anesthesia. Extraction of lower erupted tooth was done under inferior alveolar nerve block. Parameters taken were onset of anesthesia, duration of anesthesia and pain. Heft-Parker visual analogue scale was taken to evaluate the pain response during procedure after every 10 minutes until complete return of sensation by probing. The Chi-square test was used to compare the pain among the groups. The continuous variables were compared among the groups by one way analysis of variance (ANOVA) followed by Tukey’s post-hoc comparison tests. The *p*-value <0.05 was considered significant.

**Results:**

The onset of tingling sensation was higher among the patients of group C (1.53±0.57) than group B (1.50±0.58), group D (1.48±0.51) and group A (1.45±0.62) but difference among the groups was statistically insignificant (*p* >0.05). The total time in return of sensation was higher among the patients of group C (70.30±4.34) than group A (65.94±3.45), group B (62.23±7.47) and group D (47.70±8.04) but difference among the groups was found to be statistically significant (*p*=0.0001). There was no significant (*p* >0.05) difference in the pain at baseline and at start. No pain was found among all the patients from 10 minutes to subsequent time intervals.

**Conclusions:**

Mannitol was effective in increasing the efficacy of lidocaine as an adjuvant to local anesthetic solution in inferior alveolar nerve block.

** Key words:**Inferior alveolar nerve block, lidocaine, local anesthesia, mannitol.

## Introduction

Since the beginning of time surgeons have been searching for the substances that can be used to make procedures painless. Such substances are the anesthetics. The most commonly used local anesthetic in dentistry is lidocaine. An ideal solution is where profound anesthesia is achieved and maintained throughout the procedure and followed by early and complete recovery of sensation. For decades, mannitol is the most widely used agent in the management of raised intracranial pressure, as a renal protective agent in patient with high risk of developing renal failure, and as prophylaxis against acute renal failure surgeries. A hyperosmolar solution can cause a transient and artificial opening of perineurium; this enhances the permeability for macromolecules and /or ions. The hyperosmolar solution of mannitol did not induce an inflammatory cell infiltrate when the tissue was examined histologically (1). There is paucity of literature mentioned use of mannitol with local anesthetic solution. Our study focuses on this aspect of combination of mannitol with local anesthesia and its possible use in oral and maxillofacial surgery. The aims and objectives of the study were to assess whether 0.5 M of mannitol could increase the efficacy of lidocaine with or without epinephrine in inferior alveolar nerve block.

## Material and Methods

The present study comprised of 120 patients requiring extraction of erupted mandibular posterior teeth. Individuals between the ages of 18 and 35 years who have no allergy to mannitol and lidocaine were included in the study. Subjects allergic to mannitol or lidocaine or with any underlying systemic disease or history of significant medication that would alter pain perception and pregnant and lactating mothers were excluded from the study.

The patients were randomly divided into four groups, 30 patients in each group. Group A was administered 2% lidocaine with 1:80000 epinephrine; group B, 2% lidocaine with 1:80000 epinephrine and 0.5 M mannitol; group C, 2% lidocaine and 0.5 M mannitol; and group D (control group), 2% lidocaine for achieving local anesthesia

Type of anesthesia included in the study was inferior alveolar nerve block and surgical procedure included was lower erupted tooth indicated for extraction. Inferior alveolar nerve block and extraction were performed by experienced dental surgeon. Parameters taken were onset of anesthesia, duration of anesthesia and pain. Heft-Parker visual analogue scale was taken to evaluate the pain response during procedure.

-Method of the study:

Before treatment, surgical technique was briefed to patient and verbal and written consent was taken. This study was approved by the Institutional Ethical Committee for human subjects and also conducted in accordance with the Declaration of Helsinki in 1975, as revised in 2000. Subjects were administered inferior alveolar nerve block and extractions were done.

The molarity of the mannitol solution used in this study would be 0.5 M. The following calculations were utilized to make 0.5 M of mannitol; Molarity (M), or the molar concentration is defined as the ratio between the numbers of Moles of a solute per liter of solution. Molecular wt. of mannitol = 182.172, commercially available 20 % mannitol will contain (200/182.172 = 1.098 M) of the mannitol, i.e. approx. 1 Mole in a bottle. To make it 0.5 Mole the equal dilution of the solution should be done using normal saline/ sterile water, 1 ml of 20% mannitol solution + 1 ml of normal saline = 0.5 M of mannitol.

For group A, 1.5 ml of 2% lidocaine with 1:80000 epinephrine and for group D (control group), 1.5 ml of 2% lidocaine were withdrawn from standard dental cartridge into a 3.0 ml Luer-Lok disposable syringe with 24 gauage X 25 mm needle. For group B, 1.5 ml of 2% lidocaine with 1:80000 epinephrine and for group C, 1.5 ml of 2% lidocaine were withdrawn from standard dental cartridge into a 3.0 ml Luer-Lok disposable syringe with 24 gauage X 25 mm needle followed by addition of 0.9 ml of 0.5 M mannitol into both syringes. Each patient received only one cartridge in each group. Following the administration of the anesthetic solution, as per scheduled inferior alveolar nerve block, the time of injection and the time of onset of the tingling sensation on the lower lip was recorded. Pain response was assessed which corresponds to no pain, mild pain, moderate pain and severe pain after the interval of 10 minutes until the return of complete sensation on probing. The difference between the time of onset of tingling sensation on lower lip and the time of return of sensation on probing also recorded to determine the duration of the anesthesia for each group.

-Statistical tools used:

The results are presented in mean ±SD and percentage. The Chi-square test was used to compare the pain among the groups. The continuous variables were compared among the groups by one way analysis of variance (ANOVA) followed by Tukey’s post-hoc comparison tests. The *p*-value<0.05 was considered significant. All the analysis was carried out on SPSS 16.0 version (Chicago, Inc., USA).

## Results

-Onset of action (Tingling sensation on lower lip)

**Figure 1:**
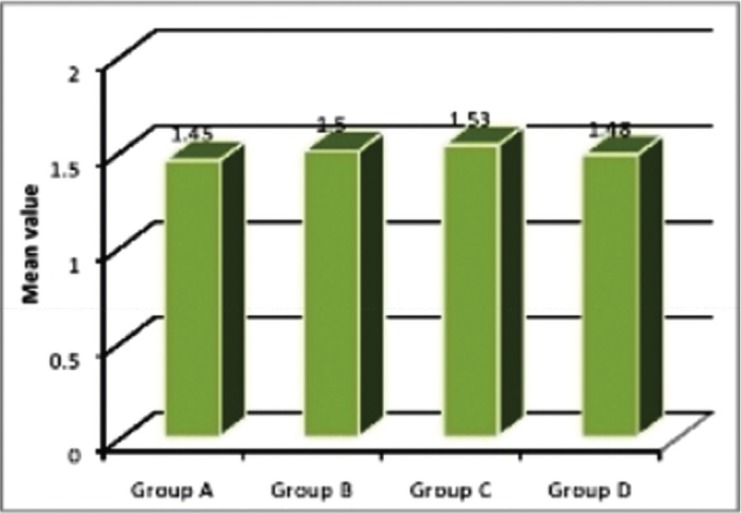
Comparison of onset of tingling on lower lip among the groups.

The onset of tingling sensation was higher among the patients of group C (1.53±0.57) than group B (1.50±0.58), group D (1.48±0.51) and group A (1.45±0.62). However, the difference was found to be statistically insignificant (*p*>0.05) among the groups ( Table 1, Fig. 1).

**Table 1:**
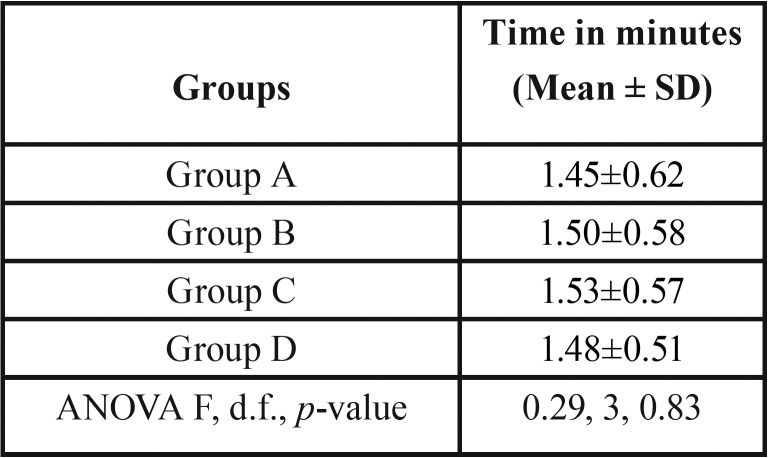
Comparison of onset of tingling on lower lip among the groups.

-Duration of action (time taken for sensation to return)

**Figure 2:**
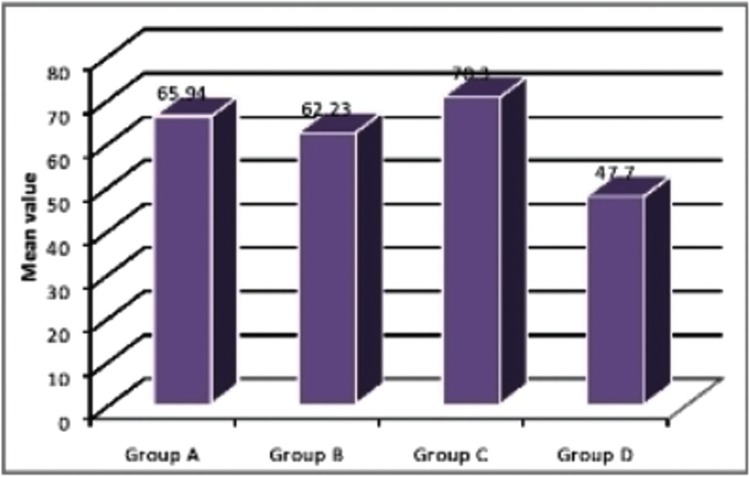
Comparison of total time in return of sensation among the groups.

The total time in return of sensation was higher among the patients of group C (70.30±4.34) than group A (65.94±3.45), group B (62.23±7.47) and group D (47.70±8.04). The difference was found to be statistically significant (*p*=0.0001) among the groups ( Table 2, Fig. 2).

**Table 2:**
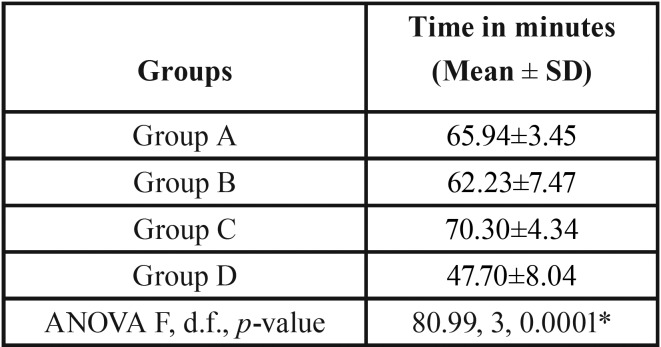
Comparison of total time in return of sensation among the groups.

The post-hoc comparison tests showed that the total time in return of sensation was significantly different between group A and group C (*p*=0.025) as well as with group D (*p*=0.0001). A significant (*p*=0.0001) difference was also found between group B and group C and group D. The difference between group C and group D was also found to be statistically significant (*p*=0.0001) ( Table 3).

**Table 3:**
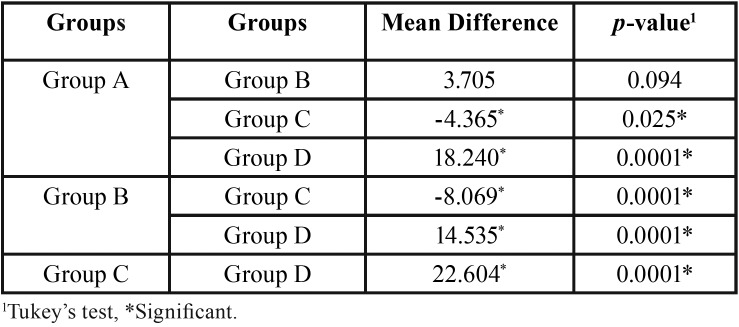
Post-hoc comparison tests of total time in return of sensation among the groups.

-Pain

There was no significant (*p*>0.05) difference in the pain at baseline and at start. No pain was found among all the patients from 10 minutes to subsequent time intervals ( Table 4).

**Table 4:**
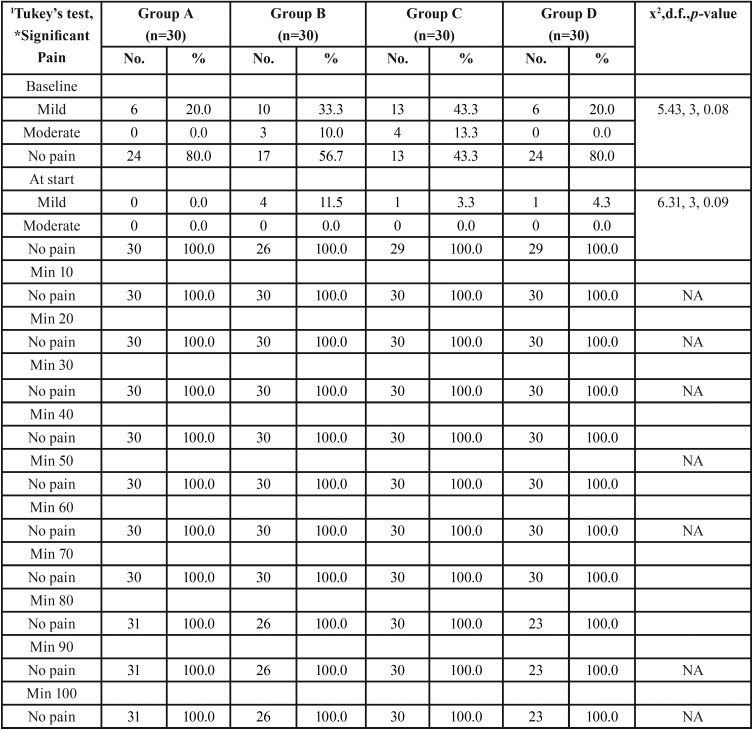
Comparison of pain among the groups at different time intervals.

## Discussion

In the present study mannitol was used, because it is inert and we would not expect it to chemically combine or react with lidocaine or epinephrine (1). The pH values of the lidocaine and lidocaine/ mannitol formulations were similar, 6.60 and 6.672, respectively. It is unlikely that pH caused differences in success rates of the inferior alveolar nerve block (2).

In current study we found that Mannitol was effective in increasing the efficacy of the local anesthetic solution in inferior alveolar nerve block. Antonijevic *et al.* (1) found that a hyperosmolar solution of mannitol caused a transient, artificial opening of the perineurium. This is in support to the mechanism of mannitol. Our results would support the findings of Antonijevic *et al.* (1) for an inferior alveolar nerve block, and that it probably affect the duration of the anesthesia of local anesthetics.

For this study we chose a hyperosmolar solution of mannitol since Antonijevic *et al.* (1) demonstrated that, the hyperosmolar solution of mannitol did not induce an inflammatory cell infiltrate when the tissues were examined under horseradish peroxidase histochemistry in male Wistar rats. Present study also supports the study done by Matsuka and Spigelman (3) on male Sprague - Dawley rats and stated that, hyperosmolar solutions selectively block action potential in rat myelinated sensory fibres; they also reported that the hyperosmolar solutions produce a selective block of signal propagation in myelinated sensory A-fibres. Their results on the action of the hyperosmolar solutions also support our study and it might also affect the depth of the local anesthesia. 

In our study we found that the mannitol was effective in increasing the efficacy of the local anesthetic solutions in inferior alveolar nerve block. The result of our study is in accordance to the studies done by Wolf *et al.* (4), Thimmaiah *et al.* (5) and Smith *et al.* (2). Smith *et al.* (2) in their study reported that, the posterior teeth had higher value of the total pulpal anesthesia than anterior teeth and they tested mandibular anterior and posterior teeth with a pulp tester. They tested teeth at every 4 minute cycle for 60 minutes after solution deposition, for postoperative pain. While in our study, we recorded the time of injection and the onset of the tingling sensation on lower lip, to compare the time of onset of action among the four groups. After comparison the onset of tingling was higher among the patients of group C (1.53±0.57) than group B (1.50±0.58), group D (1.48±0.51) and group A (1.45±0.62). However, this difference was found to be statistically insignificant (*p*>0.05); suggesting that time of onset of anesthesia was irrespective of the type of solution that were used in this study.

The duration of action of local anesthetic solution in different groups was in following order: patients of group C (70.30±4.34) then group A (65.94±3.45), group B (62.23±7.47) and group D (47.70±8.04). The difference was found to be statistically highly significant (*p*=0.0001); suggesting clearly that group C where mannitol was used with lidocaine only was the group with longest duration of action. With ANOVA value of 80.99, we couldn’t find any other relevant study with respect to the clinical use of lidocaine with mannitol, nor was much data for independent use of lidocaine without epinephrine except for known cardiac ailments or conditions like pheochromocytoma etc.

In our study, we used 0.5 M mannitol for the formulation which is less irritant and has more efficacious when used with lidocaine instead of using 0.9 M mannitol used by Talati *et al.* (6) which was more irritant after the injection. Present study also supports the study done by Wolf *et al.* (4) and Thimmaiah *et al.* (5). Wolf *et al.* (4) used three formulations one formulation containing lidocaine and epinephrine (control group) compared with the two formulations containing lidocaine and mannitol in different volumes. While in our study the total volume of the solution was kept the same but on the different formulations. Both studies done by Wolf *et al.* (4) and Thimmaiah *et al.* (5), favor that, mannitol is probably effective in increasing the efficacy of local anesthesia. Adib Hajbaghery *et al.* (7) in their study also concluded that, adding 0.5 Mole mannitol to lidocaine with epinephrine formulations significantly improved the effectiveness in achieving a greater percentage of total pulpal anesthesia, compared with a lidocaine formulation without mannitol for inferior alveolar nerve block which is in support of our study.

Our study is contradictory to the study done by Ridenour *et al.* (9) which concluded that adding hyaluronidase to a buffered solution of lidocaine with epinephrine didn’t statistically increase the incidence of pulpal anesthesia in inferior alveolar nerve block and because of its potential tissue damaging effect; it should not be added to local anesthetic solutions for inferior alveolar nerve blocks. In our study patients administered with mannitol reported prolonged effect of the local anesthetic. There has been no complication observed with the use of mannitol in our study. The results obtained following statistical analysis shows increased efficacy of the inferior alveolar nerve block on use of 2 % lidocaine without epinephrine with 0.5 M mannitol. The study conducted showed a significant increase in the efficacy of the local anesthetic on addition of mannitol which is in perfect agreement with Smith *et al.* (2) and forms the basis of present study.

## Conclusions

In our study mannitol showed its efficiency in increasing the efficacy of lidocaine inferior alveolar nerve block even at low dose with desired duration of action. This substance has the potential to be studied for its further application in the maxillofacial surgical field.
